# The effects of isobaric and hyperbaric bupivacaine on maternal hemodynamic changes post spinal anesthesia for elective cesarean delivery: A prospective cohort study

**DOI:** 10.1371/journal.pone.0226030

**Published:** 2019-12-12

**Authors:** Shamill Eanga Helill, Wossenyeleh Admasu Sahile, Ritbano Ahmed Abdo, Getahun Dendir Wolde, Hassen Mosa Halil

**Affiliations:** 1 Department of Anesthesia,College of Medicine and Health Sciences,Wachemo University, Hossana, Ethiopia; 2 Department of Anesthesia, School of Medicine, Addis Ababa University Addis, Addis Ababa, Ethiopia; 3 Department of Midwifery,College of Medicine and Health Sciences,Wachemo University, Hossana, Ethiopia; 4 Department of Anesthesia, School of Medicine, Wolaita Sodo University, Wolaita, Ethiopia; Cleveland Clinic, UNITED STATES

## Abstract

**Background:**

Spinal anesthesia is a form of regional anesthesia frequently used in various lower abdominal, orthopedic, obstetric operations such as a cesarean delivery. The most common local anesthetic used for spinal anesthesia in obstetric and non-obstetric surgery is bupivacaine which can be utilized as an isobaric or hyperbaric solution, producing differences in maternal hemodynamic changes. Against this backdrop, the study aims to compare the effects of isobaric and hyperbaric bupivacaine on maternal hemodynamic alterations after administering spinal anesthesia for elective cesarean delivery at Gandhi Memorial Hospital, Addis Ababa, Ethiopia.

**Methods:**

A hospital-based prospective cohort study design was employed for the period December 1, 2017 to January 30, 2018. A total of 100 parturient were involved, with one group exposed to isobaric bupivacaine and the other to hyperbaric bupivacaine to observe their effects on maternal hemodynamic changes post spinal anesthesia. The participants were selected through systematic random sampling. Data analysis was performed using SPSS (version 20) through descriptive statistic, independent sample t-test, Mann-Whitney U-test, Fisher’s exact test, and Chi-square test were used. P values of <0.05 was assumed as statistically significant for all tests.

**Results:**

The incidence of hypotension was found to be greater in isobaric than hyperbaric groups (82% vs. 60% respectively; *p =* 0.015). No statistical significant differences were found in mean arterial pressure value at baseline, but, statistically significant changes were observed among the groups *(p <*0.05) at all study timing after spinal anesthesia, except at 30^th^min. No statistically significant differences were seen in the mean heart rate variability after spinal anesthesia at all periods, except the 15th minute (*p =* 0.033). A greater rate of vasopressor was used in the isobaric group as compared to the hyperbaric group (36% vs. 14% respectively; p = 0.011).

**Conclusion:**

Baricity is a significant factor in maternal hemodynamic changes in the parturient for elective cesarean section. Isobaric bupivacaine produces greater change in blood pressure and incidence of hypotension and entails a greater vasopressor requirement than hyperbaric bupivacaine after spinal anesthesia for elective cesarean section.

## Introduction

Spinal anesthesia (SA) is a form of regional anesthesia (RA) used frequently in various lower abdominal, orthopedic, and obstetric operations, including cesarean delivery (CD) [1–4]. The most common anesthetic used for SA in obstetric and non-obstetric surgery is bupivacaine, which can be formulated as an isobaric or hyperbaric solution [5–7].

Cesarean delivery is usually performed under SA using hyperbaric bupivacaine. This has been associated with an increased incidence of severe hypotension [8]. Isobaric bupivacaine is not commonly used for SA but can be a good alternative for obstetrics due to its lower maternal hemodynamic changes [9]. Besides the volume, concentration, and dose, the baricity of the solution can affect the spinal block profile [10–13].

Baricity differences between spinal anesthetic solutions are thought to affect hemodynamic parameters and distribution within the subarachnoid space, which may, in turn, affect onset, extent, and duration of sensory block as well as side effects [14].

It is commonly believed that hyperbaric solutions are more suitable to reach the higher thoracic dermatomes as opposed to their plain equivalents [15]. The aim of all anesthesia professionals is to perform SA with the lowest deviation in blood pressure and heart rate, assuming that the patient is hemodynamically normal preoperatively. In doing so, RA of various baricity are used [16].

Hypotension is the most common effect of neuraxial anesthesia, mainly in obstetric surgery. The prevalence of hypotension in obstetric patients under SA, particularly in CDs, is 80–90% [8]. Hypotension causes unpleasant symptoms such as nausea and vomiting, bradycardia, unconsciousness, respiratory depression, and cardiac arrest in mothers who undergo CD. In severe and prolonged conditions, it leads to impairment of uterine perfusion and, ultimately, fetal acidosis and neonatal depression [17, 18].

Factors that increase the risk of hypotension include patient factors (advanced age, female sex, pregnancy, obesity, diabetes mellitus, hypertension, anemia) and technical factors such as a block level at or above T5, the baricity of the drugs, use of opioids as premedication, and high RA dosages [19, 20].

Published data shows that various measures have been taken to reduce spinal-induced hypotension in parturients undergoing elective CD, such as left-lateral tilt; IV fluid preloading or co-loading; leg elevation; and prophylactic use of vasopressors, but the incidence of hypotension is still high [21, 22]. Additional studies have been recommended to determine the relative effect of the baricity of regional anesthetics on spinal block characteristics, especially in parturients. To the best of our knowledge, there is no published research found in Ethiopia that shows the effects of isobaric and hyperbaric bupivacaine on maternal hemodynamic changes. Therefore, the study aims to compare the effects of isobaric and hyperbaric bupivacaine on maternal hemodynamic alterations after administering spinal anesthesia for elective cesarean delivery at Gandhi memorial hospital, Addis Ababa, Ethiopia.

## Methods and materials

A hospital-based prospective cohort study design was employed for the period December 1, 2017 to January 30, 2018 at Gandhi memorial hospital. The source population consisted of pregnant mothers who underwent an elective CD during the study period. Study population consisted of selected pregnant mothers who underwent an elective CD during the study period. The inclusion criteria were: all women underwent an elective caesarean delivery during the study period. On the other, mothers with systemic and psychological disorders, mother with the pregnancy complications, emergency CD, failed spinal block, any contraindications for spinal anesthesia and allergy to RA were excluded from the study.

Pregnant mothers who underwent an elective CD with isobaric comprised the exposed group and those who underwent with Hyperbaric Bupivacaine comprised the unexposed group. The sample size was computed by using the double population proportion formula. The following assumptions were used to estimate the sample size; the incidence of hypotension was taken from the preliminary data from Iran (42% in the unexposed group and 70% in the exposed group) [23], with a 95% confidence interval, a 5% desired precision and 80% power, the sample size was 100 (50 per group). A systematic random sampling method was used to select study participants from each group. From all scheduled parturient who fulfilled the inclusion criteria to undergo caesarean section on the operation list, the samples were selected skipping every 2 interval. The first parturient was selected by a lottery method from the first two schedules. Based on the decision of anaesthetists, the selected parturient were placed in either group until the required sample size was filled.

Data were collected using pretested structured questionnaires. Anaesthetists, two with bachelor’s degree and one with a master’s degree, were hired for the data collection and supervision. Preoperative evaluation was done a day before operation to keep NPO, to fulfil necessary investigations based on historical findings, and to perform a physical examination via hospital anaesthetists. The functionality of the operation room equipment was checked and prepared with the necessary drugs early in the morning. Before the parturient entered the operation room, a chart review including socio-demographic identification, maternal characteristics, and indication for CD was identified by on-duty anaesthetists assigned per hospital programme. All parturient were preloaded with 0.9% NS 10–20 ml/kg. Premedication ceftriaxone 1gm and metoclopramide 10 mg IV were given upon arrival in the waiting area for the operation room.

The operation room routine included standard monitoring with non-invasive blood pressure, electrocardiogram (ECG), and pulse oxymetry monitoring. The baseline blood pressure and heart rate were measured five minutes before administration of the spinal anaesthesia. Under all aseptic conditions, spinal anaesthesia was instituted in the sitting position using the anterior superior iliac crest as a surface landmark with a 25-G pencil-point spinal needle at L3-4 inter-space level. After the correct needle placement, as identified by the free flow of CSF, 12.5mg of bupivacaine was given over a period of ten seconds.

Immediately after the spinal injection, the parturients were gently assisted to lie in supine position kept at 15^0^ left uterine displacement. Patients were followed for 30 minutes after SA administration. Data collectors recorded vital signs every 5 minutes throughout the surgery and success of spinal anesthesia was assessed by sympathetic block, reduction in motor function. The level of sensory blockade was assessed by applying iced gauzes on the mother’s skin and documented. Parturients who developed hypotension and not enabled to correct with fluid and position, ephedrine 5mg IV bolus were used for treatment of hypotension. **Hypotension:** In this study, hypotension was defined as a 25% decrease in systolic blood pressure from pre-anesthetic value after the spinal anesthesia administration.

The quality of data maintained through, a questionnaires was pre-tested on 5% of the sample size at black lion specialized hospital, and necessary modifications were made based on the nature of gaps identified in the questionnaire. Additionally, data collectors and supervisors were oriented for a day by the investigators on the content of the questionnaire. Moreover, the supervisors and the investigators closely followed the day-to-day data collection process during the pretest and the actual data collection.

### Data processing and analysis

Data were entered and analysed using SPSS version 20.0. Descriptive statistics (i.e., frequency, percentage, median, mean, and standard deviation) were applied to describe the pattern of the data. Furthermore, the normality of the quantitative data was evaluated using the Shapiro-Wilk and Kolmogrove-Smirnov test, and the homogeneity by the Levanes test of equality of variance. Independent samples t-test, Mann-Whitney U-test, chi-square test and Fisher’s exact test were used. P values of <0.05 were assumed as statistically significant for all tests.

### Ethics approval and consent to participate

The ethical clearance letter was obtained from Institutional Review Board of Addis Ababa University, department of anesthesia research ethical committee. Additionally, permission was obtained from the Gandhi memorial hospital administration. Finally, written informed consent was obtained from each participant, which has been included in the study during the data collection time, after explaining the objectives of the study to them. Furthermore, confidentiality was maintained through not asking personal identifiers like name and address.

## Results

### Demographic, perioperative characteristics of parturients

A total of 100 women who had undergone CD were included in the study. Within this group, 50 parturients took isobaric and 50 parturients took hyperbaric. No statistically significant differences were observed among the groups with respect to age, weight, height, BMI, ASA status, parity, gestational age, indication for CD, sensory block level, amount of fluids used, amount of blood loss, baseline SBP and baseline HR. A majority of parturients had undergone an elective cesarean with an indication of previous CD (56% in the hyperbaric and 62% in the isobaric groups) **(Table 1)**.

**Table 1:** Demographic data, perioperative variables among groups at Gandhi Memorial Hospital, Addis Ababa, Ethiopia, 2018.

Variable		Hyperbaric group(n = 50)	Isobaric group(n = 50)	P value
Age(yr)		28.60±3.39	29.72±3.67	0.11
Wight (kg)		66.32±5.76	68.74±9.62	0.13
Height (cm)		162.5 (5)	160 (5)	0.205
BMI(kg/m^2^)		25.02±2.129	25.818±3.199	0.145
ASA status	I	33 (66%)	35 (70%)	0.668
	II	17 (34%)	15 (30%)	
Parity	Nullipara	5 (10%)	7 (14%)	0.780
	I	22 (44%)	18 (36)	
	II	20 (40%)	23 (46%)	
	III	3 (6%)	2 (4%)	
Gestational age(wk)		39.75±1.73	39.42±1.39	0.294
Indication for CS	Previous CS	28 (56%)	31 (62%)	0.07
	Malpresentation	12 (24%)	6 (12%)	
	CPD	8 (16%)	2 (4%)	
	Others	2 (4%)	11 (22%)	
Oxytocine dose(Iu)		20 (0)	20 (0)	0.767
Total fluid used (ml)		2750(1000)	2500 (1000)	0.458
Amount of blood loss (ml)		500 (150)	500 (163)	0.570
Baseline SBP		126.72±10.37	125.26±12.322	0.523
Baseline HR		96.04±12.665	92.12±11.783	0.112

CPD: cephalopelvic disproportion

### Hemodynamic characteristics of parturients

The incidence of systolic hypotension after the administration of spinal anesthesia was 62% [95% CI, 46%–74%] in the hyperbaric group and 82% in the isobaric group with X^2^ = 5.90. Statistically significant differences were observed between the groups (p = 0.015). While a greater incidence of hypotension was seen in the isobaric than in the hyperbaric group, there were no statistical differences in mean MAP value at baseline. After spinal anesthesia was administered, statistically significant changes were observed among the groups (p < 0.05) during all the study timings except at the 30^th^ minute. Decreased MAP was more prevalent in the isobaric than the hyperbaric group from the 5^th^ to the 25^th^ minutes **(S1 Fig).**

No statistically significant differences was observed in the mean HR variability after the administration of spinal anesthesia at all the study periods between the groups, except at the 15^th^ minute. A significantly greater heart rate variability was recorded in the isobaric group at the 15^th^ minute than the hyperbaric group after the administration of spinal anesthesia as compared to the baseline values (p = 0.033). Since none of the clients developed bradycardia, no supplementary atropine was required **(Table 2).**

**Table 2:** Change in HR in first 30 minute among groups at Gandhi Memorial Hospital, Addis Ababa, Ethiopia, 2018.

Time in minute	Hyperbaric group (n = 50)	Isobaric group (n = 50)	P value
Baseline	96.04±12.665	92.12±11.783	0.112
5	96.36±14.62	91.10±15.72	0.086
10	95.16±14.33	90.32±15.47	0.108
15	95.14±12.87	89.31±14.09	0.033
20	94.04±13.42	88.88±15.16	0.075
25	94.08±12.87	89.82±12.79	0.1
30	92.38±12.22	89.9±11.94	0.307

### The effect of isobaric and hyperbaric bupivacaine on the level of sensory blockage

The levels of sensory block among the groups were observed, and a majority of the clients had T8 sensory block level in the hyperbaric group (64%) and T6 sensory block level in the isobaric group (62%). A sensory block level of T4 and above was observed in 3 clients from the isobaric group (6%) and none in the hyperbaric group. T10 sensory blockage level was observed in 6% of the isobaric and in 2% of the hyperbaric groups. There were statistically significant differences in the level of sensory blockage among groups (p = 0.001) **(S2 Fig).**

### Rate of vasopressor consumption between the groups and intra-operative maternal complications

The rate of ephedrine requirement was relatively greater in the isobaric group 18 (36%) than the hyperbaric group 7 (14%) for the treatment of hypotension and it is statistically significant (p = 0.011). Nausea and vomiting were the most common maternal complications, which accounted for 44% in isobaric and 20% in hyperbaric groups of all complications. Relatively higher maternal complications were observed in with respect to nausea and vomiting, light headedness and respiratory depression in isobaric group. It was statistically significant between the groups (p = 0.001).

## Discussion

In this study, the incidence of hypotension was found to be greater in the isobaric than in the hyperbaric group (82% vs. 60%; *p =* 0.015). Statistically significant differences were observed between groups. Similar findings were also reported in a study done in Pakistan [24], and Turk [25]. In contrast, this study found a higher incidence of hypotension in isobaric than in hyperbaric groups compared to studies carried out in, Iran [23], United Kingdom [26], and Wales [27]. These differences might be due to difference in position, block height, spine curvature, socio-demographic characteristics and experiences of anesthetists.

There was no statistical significant differences in mean MAP value at baseline, but after spinal anesthesia statistically significant changes were observed between the groups at all study timing, except at 30^th^ minute. Decreased MAP in this study was observed more in isobaric than in hyperbaric groups from 5 to 25 minutes. In contrast, other studies conducted in Bosnia and Herzegovina[7] and Wales [27] revealed a greater reduction in MAP in hyperbaric than in isobaric groups. These differences might be due to the position and height of parturient, volume of local anesthetics used, the possibility of dehydration, and the amount of vasoactive drugs used.

In addition, a significantly higher heart rate (HR) variability was recorded in the isobaric group at the 15^th^ minute than in the hyperbaric group with respect to baseline values. This finding is in contrast with a study conducted in Indonesia [28] that reported no significant differences in the HR variability between groups. On the other hand, our result is inconsistent with a study done in Bosnia and Herzegovina [7] that reported pulse frequency decreased significantly in hyperbaric groups in comparison to isobaric groups.This difference might be due to blockage of sympathetic accelerator fibers and high level of sensory blockage.

This study revealed a higher level of sensory blockage in the isobaric groups. This is supported by researches done in Indonesia [28]. This was in contrast to what was observed in Iran[23] which showed a lower sensory block level in isobaric groups. The observed difference might have been due to the fact that adopting supine position, even with left lateral tilt, causes inferior vena cava compression, which in turn results in an engorgement of the epidural venous plexus. The consequent dural sac compression may facilitate bulk movement of drugs injected into the CSF and could explain the cephalad progression of the isobaric bupivacaine.

In this study, a greater rate of ephedrine was required in isobaric groups. However, in contrast to what was found in Wales [27] and Iran [23], an ephedrine requirement was not found to be greater in the hyperbaric group in this study.

Nausea and vomiting were the most common maternal complications, which accounted for 44% in isobaric and 20% in hyperbaric groups of all complications. The occurrence of nausea and vomiting might be secondary to hypotension, which was effectively reversed with fluid administration and ephedrine as a vasopressor. In contrast to our result, studies done in the United Kingdom and Pakistan found insignificant differences in the incidence of nausea and vomiting [10, 24]. This might be due to difference in the incidence of hypotension, vagal hyperactivity, visceral pain, utero-tonic agents, and sympathectomy.

**Strength of the study**: Study participants were selected using probability sampling method to ensure its representatives and different approaches were used to maintain the quality of data. No loss of parturients that resulted in missing data to follow up on. **Limitations of the study** are the inaccessibility of invasive arterial blood pressure to measure parturient beat-to-beat systolic blood pressure. The onset and duration of sensory and motor blockage was not evaluated. The duration of hypotension could not be measured with every hypotensive episode due to noninvasive monitoring device is adjusted every 5 minutes in the hospital.

## Conclusion

Baricity is a significant factor in maternal hemodynamic changes in the parturient for elective cesarean section. Isobaric bupivacaine produces a greater change in blood pressure and incidence of hypotension and entails a higher vasopressor requirement than hyperbaric bupivacaine after spinal anesthesia for elective cesarean section.

## Supporting information

S1 Fig Change in mean MAP in first 30 minute after spinal anesthesia among groups at Gandhi Memorial Hospital, Addis Ababa, Ethiopia, 2018. (TIF)

S2 Fig Level of sensory block after spinal anesthesia among groups at Gandhi Memorial Hospital, Addis Ababa, Ethiopia, 2018. (TIF)

S1 File Consent form and questionnaire. (DOCX)

S2 File SPSS. (SAV)

## Abbreviations

ASA: American Society of Anesthesiologist
BMI: Body Mass Index
BSc: Bachelor of Science
CPD: Cephalopelvic Disproportion
CD: Cesarean delivery
CSF: Cerebrospinal Fluid
LA: Local Anesthetics
MAP: Mean Arterial Blood Pressure
MSc: Masters of Science
PI: Principal Investigator
SA: Spinal Anesthesia
SBP: Systolic Blood Pressure
