# Supplementary figures and images for: The effects of isobaric and hyperbaric bupivacaine on maternal hemodynamic changes post spinal anesthesia for elective cesarean delivery: A prospective cohort study
Source: PLoS One. 2019 Dec 12;14(12):e0226030. doi: 10.1371/journal.pone.0226030 (PMC6907792; doi:10.1371/journal.pone.0226030)

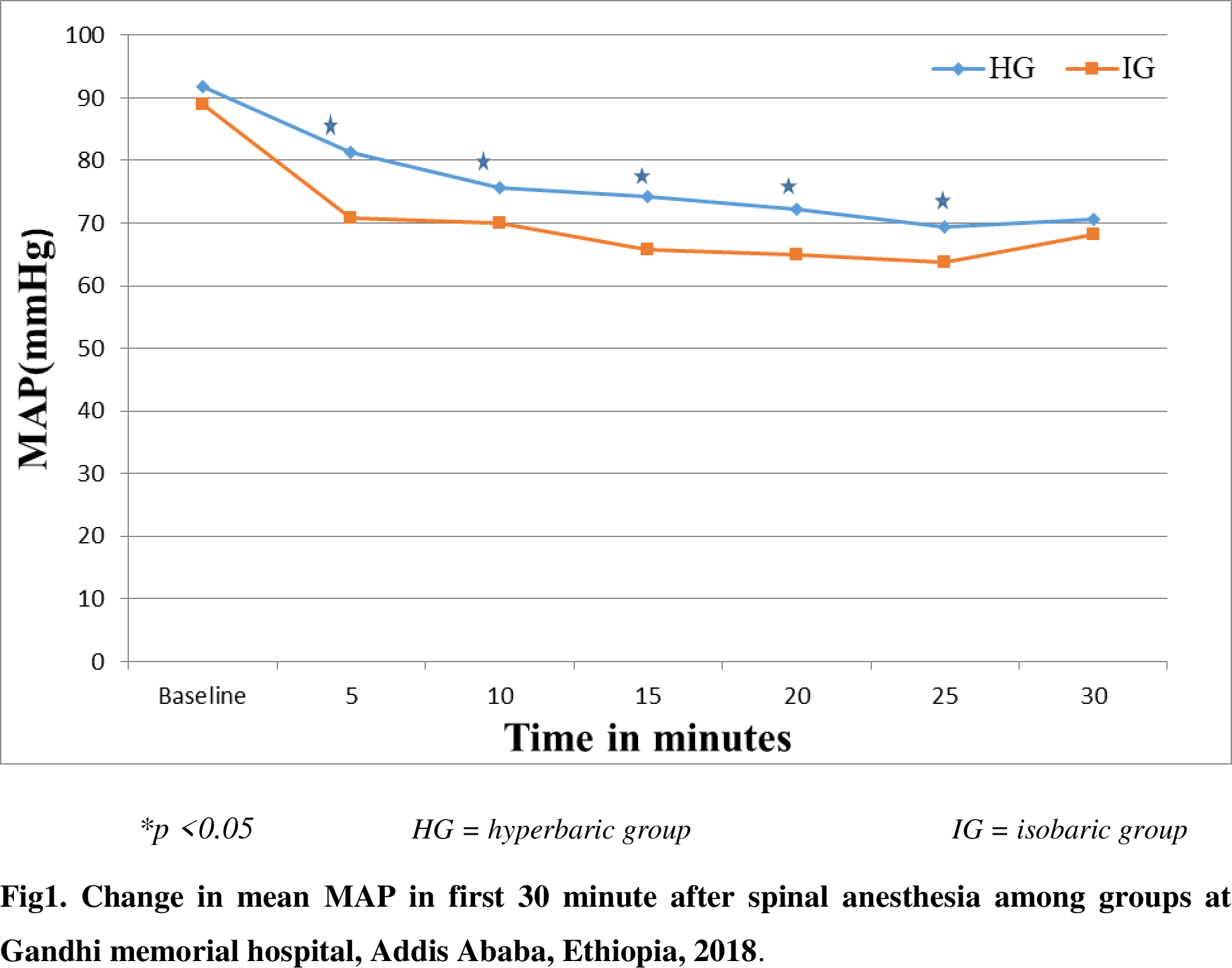

Supplement: S1 Fig — (TIF) [file pone.0226030.s001.tif]

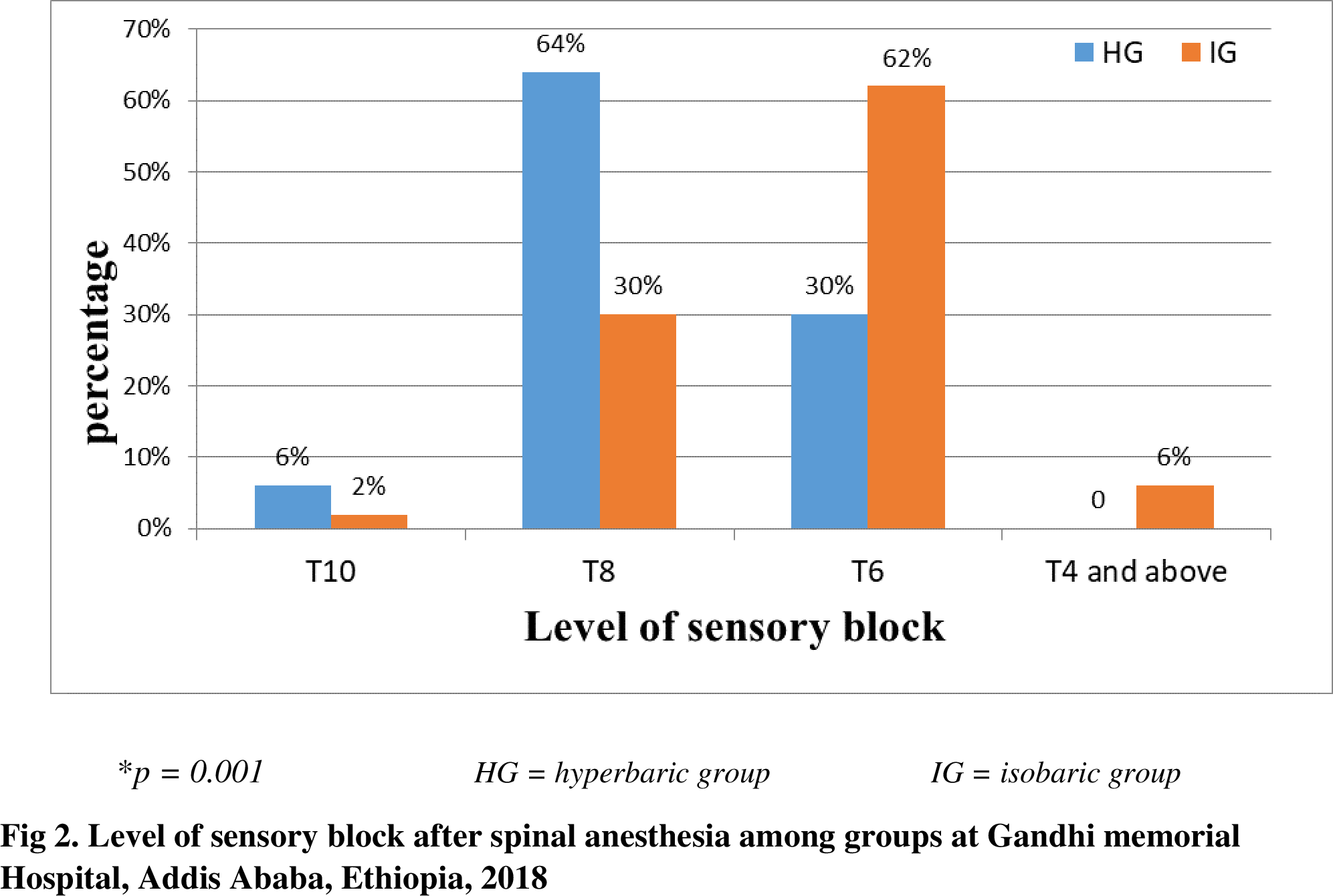

Supplement: S2 Fig — (TIF) [file pone.0226030.s002.tif]
