# Supplementary material for: The effects of isobaric and hyperbaric bupivacaine on maternal hemodynamic changes post spinal anesthesia for elective cesarean delivery: A prospective cohort study
Source: PLoS One. 2019 Dec 12;14(12):e0226030. doi: 10.1371/journal.pone.0226030 (PMC6907792; doi:10.1371/journal.pone.0226030)
Supplement: S1 File — (DOCX) [file pone.0226030.s003.docx]

# Annex-II: Consent form (English version)

Hello-Dear Participant!
My name is _______________________________________________________________. I
am a researcher and am attending postgraduate program in the field of Anesthesiology at
Addis Ababa University. I am going to conduct a research on comparing the effects of isobaric and hyperbaric bupivacaine on maternal hemodynamic changes after Spinal Anesthesia on elective cesarean sections from December 1, 2017 to January 30, 2018 at Gandhi memorial hospital.
The information going to be obtained will help the government and other responsible bodies to reduce the incidence of hypotension which helps to reduce maternal and neonatal morbidity and mortality. Your participation is very valuable for the success of this project.
Also be mindful that whatever we will get here is for research purposes only and the information
will not be used by any other person apart from this research and therefore, confidentiality can be guaranteed. However, your names will not be mentioned or be attached to anything that you say. If you have anything to ask contact data collectors and supervisors available there.

Do you want to continue yes------------- No---------- (Thank you in advance for your help!)

Name and contact address of investigator

Shamill Eanga, E-mail: [eangashamill67@gmail.com](mailto:eangashamill67@gmail.com) Cell phone: +251 912-750-725.

# Annex IV: Questionnaires

**Identification card no.** ------------------------

| S.No | Question | Response | | Code | Skip pattern |
| --- | --- | --- | --- | --- | --- |
| 101 | Age | |  |  |  |
| 102 | Weight | |  |  |  |
| 103 | Height | |  |  |  |
| 104 | Body mass index (BMI) | |  |  |  |

**Part I Demographic characteristics**

**Part II preoperative Assessment**

| S.No | Question | Response | | Code | Skip pattern |
| --- | --- | --- | --- | --- | --- |
| 201 | Admission diagnosis | |  |  |  |
| 202 | Parity | | Gravida ________ |  |  |
|  |  |  | Para ________ |  |  |
| 203 | Gestational age in weeks | |  |  |  |
| 204 | Indication of C/S | | Previous C/S | 1 |  |
|  |  |  | CPD | 2 |  |
|  |  |  | Others specify | 3 |  |
| 205 | Maternal hemoglobin level | |  |  |  |
| 206 | Premedication | | Metoclopramide | 1 |  |
|  |  | | Cimetidine | 2 |  |
|  |  |  | Other specify | 3 |  |
| 207 | Baseline blood pressure(MAP) | | ____/____mmHg (_____) |  |  |
| 208 | Baseline heart rate | | ______(bpm) |  |  |
| 209 | Baseline RR& Spo2 | | _______breath/min & ________% |  |  |

**Part III: Intra-operative Assessment**

| S.No | Question | Response | Code | Skip pattern |
| --- | --- | --- | --- | --- |
| 301 | baricity of bupivacaine administred | Isobaric bupivacaine | 1 |  |
|  |  | Hyperbaric bupivacaine | 2 |  |
| 302 | Dose of local anesthetics | ________mg |  |  |
| 304 | Local anesthetics administered time |  |  |  |
| 305 | Level of sensory blockage | T_10_ | 1 |  |
|  |  | T_8_ | 2 |  |
|  |  | T_6_ | 3 |  |
|  |  | T_4_ and above | 4 |  |
| 306 | Skin incision time |  |  |  |
| 307 | Delivery time |  |  |  |
| 308 | Physician status | Specialist | 1 |  |
|  |  | Resident | 2 |  |
| 309 | Type of utero-tonic agent used | Oxytocine | 1 |  |
|  |  | Ergometrine | 2 |  |
| 310 | Maternal complications | Nausea and vomiting | 1 |  |
|  |  | Light headedness | 2 |  |
|  |  | Respiratory depression | 3 |  |
|  |  | Other specify | 4 |  |
| 311 | APGAR score | APGAR score at 1^st^ min  _______ |  |  |
|  |  | APGAR score at 5^th^ min  ___________ |  |  |
| 312 | Vasopressors used | Ephedrine ______(mg) | 1 |  |
|  |  | Adrenaline _______(mg) | 2 |  |
|  |  | Other specify _______ | 3 |  |
| 312 | Amount of fluid used | _________ ml |  |  |
| 313 | Total blood loss | ________ ml |  |  |

**Maternal vital sign from LA administration to the first hour of operation**

| **Vital sign** | **Baseline** | **Induction** | **Minutes** | | | | | | | | | | | |
| --- | --- | --- | --- | --- | --- | --- | --- | --- | --- | --- | --- | --- | --- | --- |
|  |  |  | **5** | **10** | **15** | **20** | **25** | **30** | **35** | **40** | **45** | **50** | **55** | **60** |
| SBP |  |  |  |  |  |  |  |  |  |  |  |  |  |  |
| DBP |  |  |  |  |  |  |  |  |  |  |  |  |  |  |
| MAP |  |  |  |  |  |  |  |  |  |  |  |  |  |  |
| HR |  |  |  |  |  |  |  |  |  |  |  |  |  |  |

Name of data collector---------------------signature---------------date---------------
Name of supervisor------------------------signature---------------date---------------
